# Eicosapentaenoic acid supplementation alleviates pruritus, enhances skin moisture, and mitigates depression in maintenance hemodialysis patients

**DOI:** 10.3389/fneph.2024.1365809

**Published:** 2024-07-30

**Authors:** Ya-ling Lin, Chia-Liang Wang, Tsay-I Chiang

**Affiliations:** ^1^ Department of Nursing, Tajen University, Pingtung, Taiwan; ^2^ Department of Nephrology, Kuang-Tien General Hospital, Taichung, Taiwan; ^3^ Department of Nursing, Hungkuang University, Taichung, Taiwan

**Keywords:** eicosapentaenoic acid, uremic pruritus, depression, skin moisture, xerosis

## Abstract

**Background:**

The objective of this study is to investigate the effects of oral supplementation with eicosapentaenoic acid (EPA) on circulating inflammatory factors, cardiometabolic parameters, skin moisturization, and the consequent symptoms of pruritus and depression in maintenance hemodialysis patients.

**Materials and methods:**

A total of 60 maintenance hemodialysis patients with severe pruritus symptoms completed this randomized, placebo-controlled study. Subjects of treatment group (n = 30) were instructed to consume 1000 mg fish oil (>900 mg EPA) and subjects of placebo group (n = 30) were instructed to consume 1000 mg soybean oil twice daily for 3 months. 5-D pruritus scoring, the Beck Depression Inventory (BDI) scale, skin moisture, serum creatinine, inflammatory factors, and cardiometabolic parameters were examined at baseline, and at the first, second, and third month post-supplementation.

**Results:**

A significantly decreased pruritus level was observed in the treatment group, whereas an opposite result was observed in the placebo group. Increased skin moisture levels on both the face and arms were observed in the treatment group, but not in the placebo group. Supplementation of EPA significantly decreased serum CRP and IL-6 levels. Significant decreases in total cholesterol (CHO), and triglycerides (TG) levels were observed; however, a decrease in high-density lipoprotein (HDL) level was observed in the treatment group. There was no change in plasma creatinine (CR) observed in both groups. A significantly decreased BDI score was observed, whereas the opposite result was observed in the placebo group. A correlational study showed that the severity of pruritus was significantly associated with skin moisture and serum CRP. The severity of pruritus was also positively correlated with the BDI score.

**Conclusion:**

Supplementation of EPA may provide multiple benefits including alleviating pruritus symptoms, addressing skin dryness, and mitigating depression in maintenance hemodialysis patients.

## 1 Introduction

Pruritus is one of the most common symptoms found in patients with advanced chronic kidney disease (CKD) or end-stage renal disease (ESRD) on dialysis. This distressing symptom adversely affects the quality of life and medical outcomes and has been regarded as an independent factor associated with mortality in dialysis patients (1). Despite improvements in dialysis facilities and therapeutic options, the prevalence of CKD-associated pruritus (CKDaP) has not significantly declined (2). CKDaP still affects 40–50% of dialysis patients (3). CKDaP manifests as a prolonged, frequent, and intensive itching sensation, ranging from general to local distribution, without any apparent skin disease or coexisting with xerosis in up to 80% patients (4, 5). Some patients may also experience superimposed complications of excoriation (4). Although various symptoms have been reported to be associated with the prevalence of CDKaP, the underlying mechanism for CKDaP is still unclear and far from elucidation.

Various pathogenic factors have been proposed to be related to CKDaP, such as xerosis, hyperparathyroidism, uremic toxin accumulation, opioid imbalance, neural dysfunction, inflammation, and immune dysregulation (2, 6). Although CKDaP has been recognized to occur through multiple mechanisms, four theories have been proposed as major contributors to the prevalence of CKDaP, including toxin deposition, peripheral neuropathy, immune system dysregulation, and opioid imbalance (7). Another etiology proposed to be related to CKDaP is the dysregulation of essential fatty acids and their metabolites derived from the cyclooxygenase and lipoxygenase pathways, as dialysis patients are well-known to have abnormal fatty acid profiles (8–10). Yerlikaya1 et al. reported that plasma omega-3 fatty acid levels were significant lower, and elevated omega-6/omega-3 fatty acid ratios were found in dialysis patients (11). Higher omega-6/omega-3 fatty acid ratios are well-known to increase risk of various chronic diseases, such as cardiovascular diseases. Moreover, higher plasma saturated fatty acid and monounsaturated fatty acid were also found in dialysis patients (11–14). Furthermore, chronic inflammation is generally found in dialysis patients (15), and anti-inflammatory treatments ameliorate CKDaP (16). Several studies have reported that supplementation with omega-3 fatty acids ameliorates CKDaP by decreasing inflammation (8, 17, 18). On the other hand, xerosis is commonly found in most dialysis patients and has been suggested as an important factor influencing CKDaP (5, 19). The severity of xerosis is positively correlated with the reducing threshold of itch sensation (20). Improving skin moisture and other dermatological conditions have been generally used to decrease xerosis and the severity of pruritus in CKDaP patients (16). Omega-3 fatty acid supplementation has been reported to improve skin dryness and pruritus (21–23).

Moreover, depression is frequently prevalent in CKD and ESRD, especially in those undergoing dialysis (24–26). The severity of CKDaP and the duration of dialysis have been reported to be positively correlated with depressive symptoms (27). Several mechanisms are suggested to explain the association between depression and CKD (26). Among them, the frequent and intense itching sensation disturbs sleep and social activity, impacting quality of life outcomes. This has been recognized as a significant factor contributing to depressive symptoms in dialysis patients (1, 27, 28). Additionally, lower levels of omega-3 fatty acids were found in dialysis patients (11). Deficiency of dietary omega-3 polyunsaturated fatty acids (PUFAs) has been suggested to be associated with mood disorders (29). Thus, supplementation of omega-3 PUFAs is suggested as a potential treatment option for CKD-related depression (29). A meta-analysis reported that supplementation of omega-3 PUFAs improves depressive symptoms (30). Although the effect of omega-3 PUFAs on the improvement of depressive symptoms has shown conflicting results or limited efficacy in some studies (31, 32), other studies reported that supplementation with omega-3 fatty acids improved CKD-related depression in dialysis patients (33, 34). Moreover, formulation with higher EPA content in omega-3 PUFAs (≥60% EPA) were reported to have clinical benefits on depressive symptoms, whereas DHA-major formulations showed no benefits (30).

In a previously reported prospective cohort study, we found that supplementation with omega-3 fatty acid (1000 mg fish oil with >900 mg EPA) ameliorated pruritus conditions. This supplementation also resulted in reduced plasma levels of inflammation markers CRP and IL-6, improved kidney function, lowered cardiovascular risk factors, and enhanced skin moisture on the face and arms during a 3-month daily supplementation period (22). In this randomized controlled study, the primary outcome of our investigation focuses on evaluating the efficacy of omega-3 fatty acids (90% EPA) and omega-6 fatty acids supplementation in improving pruritus and skin moisture among hemodialysis patients. Secondary outcomes include assessing the impact of these supplementations on additional markers such as plasma levels of inflammation markers CRP and IL-6, cardiovascular risk factors, and depression. Through this study, we aim to provide valuable insights into potential therapeutic interventions for managing CKD-associated pruritus and comorbidities in this patient population. Given the significant burden of pruritus and its adverse effects on the quality of life and medical outcomes of maintenance hemodialysis patients, there is an urgent need to explore novel therapeutic interventions that target its underlying mechanisms. In this context, the potential role of omega-3 fatty acids, particularly EPA, in modulating inflammation, improving skin health, and alleviating depressive symptoms presents a promising opportunity for intervention. However, despite growing evidence suggesting the efficacy of EPA supplementation in various health conditions, its specific impact on CKDaP and related complications remains underexplored. Therefore, this study aims to elucidate the potential benefits of EPA supplementation in alleviating pruritus, enhancing skin moisture and mitigating depression in maintenance hemodialysis patients, addressing a critical gap in the current understanding and management of this complex condition.

## 2 Subjects and methods

### 2.1 Subjects

The study was designed as a randomized, placebo-controlled trial with 2 treatment groups. The duration of the study spanned three months, during which participants were assessed at baseline and at the first, second, and third month post-supplementation. This timeframe allowed for comprehensive evaluation of the effects of omega-3 fatty acid supplementation on pruritus symptoms, skin moisture, and depression among hemodialysis patients. Volunteers of both sexes, aged over 20 years, experiencing a prevalence of pruritus symptom, and undergoing hemodialysis at least 3 times a week for over 6 months, with a dialysis adequacy (Kt/V value) of blood urea nitrogen (BUN) greater than 1.2, were recruited from a dialysis facility in Kuang Tien General Hospital, Shalu District, Taichung, Taiwan. Patients with dermatological conditions diagnosed by a dermatologist and those with allergic reactions to seafood, soybean, omega-3 fatty acids, and related products were excluded. Also excluded were patients with medical records for pruritus treatments, including topical treatments within 2 weeks, systemic treatments within 1 month, or narrowband ultraviolet B phototherapy within 6 months. Eligible patients, who had not used fish oil, were identified and approached for consent to participate in the study. The 5-D pruritus questionnaire, the Beck Depression Inventory-Second Edition (BDI-II) questionnaire, measurements of skin moisture, and blood withdrawal were requested for further analyses at baseline, and at the first, second, and third month post-supplementation.

A total of 66 volunteers were recruited, and 62 patients met the criteria as described above, with 2 patients voluntarily dropping out during the study.

### 2.2 Study intervention and administration protocol

**Figure 1:**
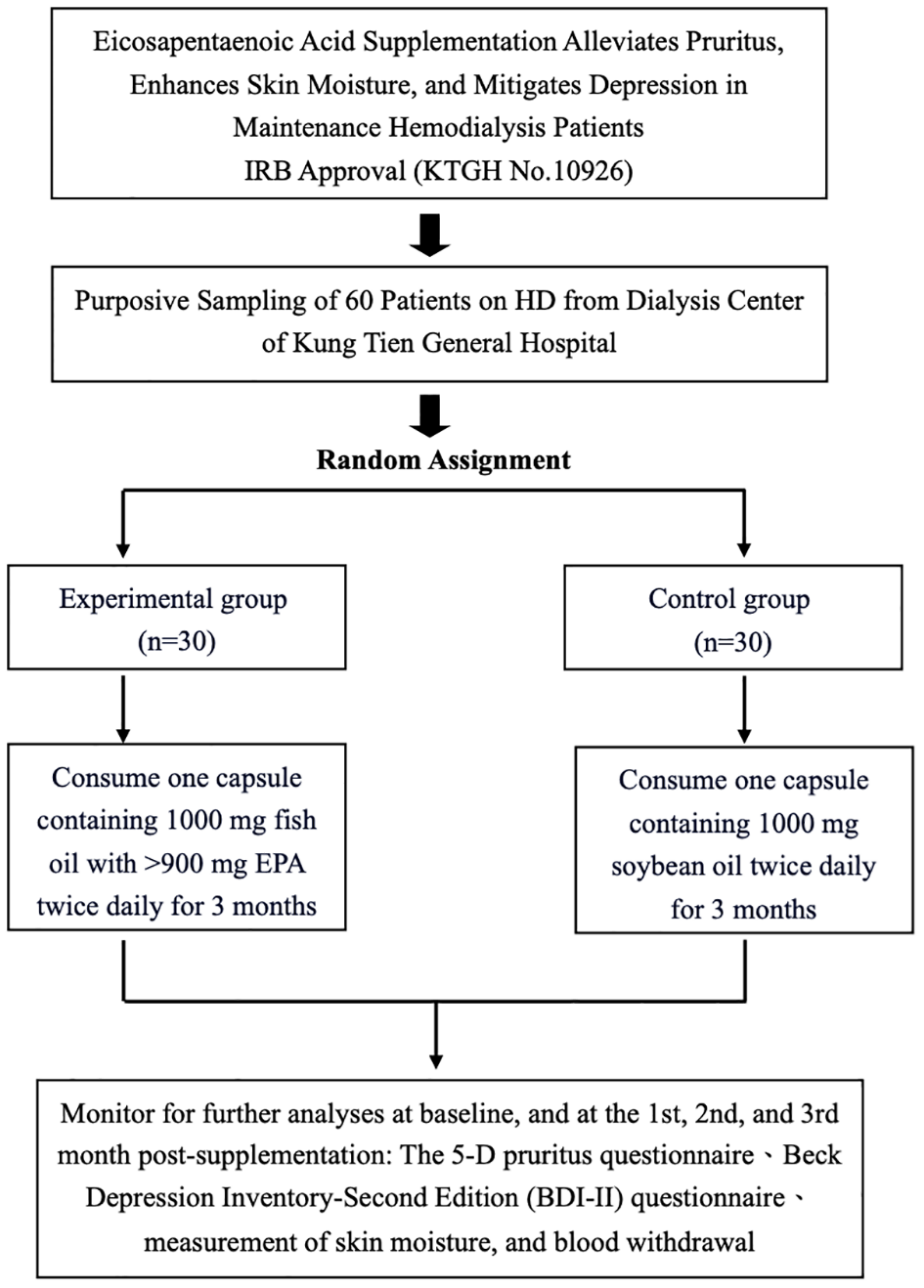
The study design.

The study design of an interventional trial investigating the effects of EPA supplementation on pruritus, skin moisture, and depression in maintenance hemodialysis patients is shown in **Figure 1**. The study received IRB approval (KTGH No.10926) and involved purposive sampling of 60 patients from the dialysis center of Kung Tien General Hospital. The participants were randomly allocated into two groups, an experimental group (n=30) and a control group (n=30). The experimental group consumed capsules containing 1000 mg of fish oil with >900 mg EPA twice daily for three months, while the control group consumed capsules containing 1000 mg of soybean oil twice daily for the same duration. Assessments were conducted at baseline and subsequently at the first, second, and third months post-supplementation. These assessments included monitoring for pruritus using a 5-D pruritus questionnaire, evaluating depression using the Beck Depression Inventory-Second Edition (BDI-II) questionnaire, measuring skin moisture, and collecting blood samples. The study aimed to investigate the potential alleviating effects of EPA supplementation on pruritus, skin dryness, and depressive symptoms in patients undergoing maintenance hemodialysis.

### 2.3 Measurement of skin moisture

Moisture levels of the face and arms were measured using Moisturemeter SC (Delfin Technologies Ltd., Kuopio, Finland) to evaluate the skin surface hydration level, as previously described (22). The device detects the tissue dielectric constant (TDC) value using a 1.25 MHz electromagnetic probe to determine conductivity in the epidermal layer, while the dry layer of the stratum corneum acts as an insulator. Average TDC values at baseline and post-supplementation were determined by constantly measuring five different sites on each surface of the face and the arms.

### 2.4 Biochemistry and interleukin-6 analysis

The collected serum from subjects at baseline and post-supplementation was measured for concentrations of c-reactive protein (CRP), creatine (CR), total cholesterol (CHO), high-density lipoprotein (HDL), low-density lipoprotein (LDL), and interleukin-6 (IL-6). Concentrations of CRP, CR, CHO, HDL, and LDL were detected by routine biochemistry analyses using clinical biochemistry systems (Beckman Coulter, Inc., Brea, CA, USA) at the clinical laboratory in the Kuang Tien General Hospital. The concentration of serum IL-6 was detected using an IL-6 ELISA kit (Biolegend, San Diego, CA, USA). All sera were stored at −80 °C until analysis.

### 2.5 Statistics

Repeated-measure ANOVA and a Chi-squared test were used to determine significant differences across each of the time points within baseline and post-supplementation. The Student t-test was used to determine significances between placebo and treatment groups. Pearson’s correlation was used to calculate the correlation coefficient and significances. Analyses were carried out using SPSS analytic software (International Business Machines Corporation, Armonk, NY, USA). A P value of <0.05 was considered statistically significant.

## 3 Results

The demographic and baseline characteristics of the participants are presented in **Table 1**. A total of 60 subjects with a hemodialysis history of over 6 months and Kt/V greater than 1.2 were randomly divided into a control group (soybean oil, n = 30) and a treatment group (EPA, n = 30). In the control group, there were 16 males and 14 females with an average age of 67.57 ± 11.57 years and an average of 6.37 ± 3.07 years on hemodialysis. In the treatment group, there were 10 males and 20 females with an average age of 66.63 ± 11.67 years and an average of 4.97 ± 3.20 years on hemodialysis. There were no significant differences in gender ratio, occupation category, education levels, age, and years on hemodialysis between the control group and the treatment group (**Table 1**).

**Table 1:** Baseline characteristics of hemodialysis patients (Mean ± SD).

Characteristic	Variable					
	Soybean oil	%	EPA	%	*X^2^*	*P_$_*
**Gender**					2.443	0.118
Male	16	53.3	10	33.3		
Female	14	46.7	20	66.7		
**Occupation category**					4.754	0.576
Unemployed (including homemakers)	14	46.7	18	60		
Commerce	9	30.0	5	16.7		
Industry	5	16.7	4	13.3		
Service industry	1	3.3	1	3.3		
Government employee	0	0.0	1	3.3		
Agriculture	0	0.0	1	3.3		
Scholars	1	3.3	0	0.0		
**Education**					2.980	0.561
Illiterate	3	10.0	1	3.3		
Primary school	5	16.7	6	20.0		
Junior high school	7	23.3	11	36.7		
Senior high school	9	30.0	9	30.0		
College/graduate	6	20.0	3	10.0		
					*t*	*P*
**Age**	67.57 ± 11.57		66.63 ± 11.67		-0.311	0.757
**Years on hemodialysis**	6.37 ± 3.07		4.97 ± 3.20		-1.730	0.089

EPA, eicosapentaenoic acid. Mean ± SD**;** X^2^, P_$_ value, Chi-square test; t value, independent samples t test, P value, unpaired t-test.

Patients in the treatment group were assigned to consume 1000 mg fish oil capsules containing 900 mg EPA twice daily. Patients in the control group received 1000 mg soybean oil capsules twice daily. There were no obvious differences in shape, color, and taste between the fish oil capsule and the soybean oil capsule. The average pruritus level presented in different parts of the body, using a 5-D itch scale questionnaire, at baseline and following supplementation with soybean oil and EPA 3 months is presented in **Table 2**. Averages of pruritus scores, skin moisture levels, depression levels using a PHQ-9 score, and biochemical profiles including CRP, IL-6, CR, CHO, HDL, LDL, and TG at baseline and following 3 months of supplementation are presented in **Table 3**. Each parameter described above was examined at baseline, and at the 1st, 2nd, and 3rd month post-supplementation.

**Table 2:** Average pruritus score presented in parts of body in hemodialysis patients following supplementation of soybean oil and EPA using a 5-D itch scale questionnaire.

Characteristic	Baseline (%)		1st Month (%)		2nd Month (%)		3rd Month (%)	
Treatment	Soybean oil	EPA	Soybean oil	EPA	Soybean oil	EPA	Soybean oil	EPA
Distribution								
Head/Scalp	10	23.33	10	16.67	16.67	6.67	10	3.33
Face	13.33	26.67	13.33	26.67	20	16.67	13.33	3.33
Chest	20	43.33	20	30	16.67	26.67	23.33	20
Abdomen	23.33	46.67	10	30	13.33	43.33	23.33	16.67
Back	46.67	80	56.67	70	66.67	53.33	63.33	46.67
Buttocks	16.67	56.67	23.33	50	20	40	26.67	30
Thighs	36.67	43.33	33.33	33.33	36.67	26.67	53.33	13.33
Lower legs	33.33	40	46.67	30	53.33	20	43.33	13.33
Top of Feet/Toes	33.33	43.33	36.67	43.33	43.33	30	40	26.67
Soles	0	6.67	6.67	3.33	6.67	3.33	3.33	0
Palms	0	3.33	3.33	0	10	3.33	3.33	3.33
Top of Hands/Fingers	26.67	30	23.33	23.33	23.33	13.33	20	10
Forearms	36.67	53.33	36.67	50	30	26.67	33.33	3.33
Upper Arms	33.33	30	23.33	26.67	23.33	13.33	36.67	0
Points of Contact with Clothing	36.67	46.67	36.67	36.67	43.33	20	40	10
Groin	40	60	43.33	63.33	60	46.67	46.67	43.33
Others	3.33	3.33	3.33	3.33	0	10	6.67	6.67

EPA, eicosapentaenoic acid.

**Table 3:** Comparison of pruritus score, biochemical profiles, moisture levels, and depression levels between placebo and EPA groups before and after supplementations in hemodialysis patients.

Characteristic	Baseline (%)	1st Month (%)	2nd Month (%)	3rd Month (%)	*P_$_*
Pruritus Score					
Soybean oil	15.70 ± 7.35	16.80 ± 6.60	18.03 ± 7.49	19.20 ± 8.34	< 0.01**
EPA	21.10 ± 6.19	18.90 ± 5.59	14.83 ± 5.59	11.90 ± 7.71	< 0.001***
*P*	0.003**	0.189	0.066	0.001**	
Moisture level (Arms; TDC value)					
Soybean oil	27.49 ± 15.97	25.98 ± 10.77	26.96 ± 15.00	25.10 ± 13.88	0.106
EPA	21.85 ± 12.52	24.02 ± 14.47	27.22 ± 16.02	31.78 ± 17.67	< 0.001***
*P*	0.136	0.597	0.97	0.117	
Moisture level (Face; TDC value)					
Soybean oil	45.13 ± 23.25	46.53 ± 25.35	44.78 ± 24.69	43.45 ± 24.24	0.329
EPA	42.40 ± 24.23	45.78 ± 27.60	50.55 ± 28.64	58.87 ± 29.79	< 0.001***
*P*	0.661	0.841	0.504	0.036*	
CRP (mg/dL)					
Soybean oil	0.77 ± 0.95	0.64 ± 0.71	0.86 ± 1.87	1.11 ± 2.00	0.604
EPA	1.87 ± 3.37	0.75 ± 0.81	0.88 ± 1.72	0.48 ± 0.60	0.025*
*P*	0.094	0.607	0.974	0.108	
IL-6 (pg/ml)					
Soybean oil	8.95 ± 12.03	19.38 ± 76.24	4.40 ± 6.35	8.41 ± 10.42	0.495
EPA	35.58 ± 96.76	11.60 ± 12.07	7.44 ± 8.80	5.93 ± 11.32	0.041*
*P*	0.14	0.622	0.1	0.386	
CR (mg/dL)					
Soybean oil	9.90 ± 1.98	10.08 ± 2.21	9.89 ± 1.99	9.71 ± 2.14	0.372
EPA	12.82 ± 9.77	11.30 ± 3.22	11.41 ± 3.76	10.96 ± 3.19	0.472
*P*	0.115	0.094	0.055	0.08	
CHO (mg/dL)					
Soybean oil	162.60 ± 36.42	158.74 ± 49.02	163.17 ± 37.71	160.47 ± 38.62	0.841
EPA	185.93 ± 43.15	168.06 ± 47.22	162.53 ± 34.07	152.07 ± 28.90	< 0.001***
*P*	0.027	0.456	0.946	0.344	
HDL (mg/dL)					
Soybean oil	38.43 ± 11.59	38.61 ± 12.13	37.62 ± 11.24	37.03 ± 10.24	0.507
EPA	37.93 ± 11.19	36.13 ± 11.42	33.23 ± 10.42	34.63 ± 13.07	< 0.001***
*P*	0.866	0.417	0.122	0.431	
LDL (mg/dL)					
Soybean oil	80.97 ± 23.56	79.45 ± 24.20	83.62 ± 24.04	87.05 ± 33.79	0.333
EPA	87.76 ± 25.67	85.44 ± 25.88	87.92 ± 28.06	82.95 ± 23.54	0.676
*P*	0.29	0.358	0.527	0.588	
TG (mg/dL)					
Soybean oil	150.43 ± 98.06	141.18 ± 70.11	127.21 ± 77.70	158.30 ± 96.68	0.247
EPA	205.34 ± 168.69	181.13 ± 126.60	159.63 ± 94.16	137.30 ± 77.08	< 0.01**
*P*	0.129	0.137	0.151	0.356	
Depression level (BDI score)					
Soybean oil	18.63 ± 9.76	19.97 ± 9.28	20.60 ± 9.70	21.33 ± 10.03	< 0.01**
EPA	25.77 ± 9.78	23.87 ± 9.91	20.67 ± 8.72	18.50 ± 9.93	< 0.001***
*P*	0.006**	0.121	0.978	0.276	

CRP, c-reactive protein; CR, creatinine; CHO, total cholesterol; HDL, high-density lipoprotein; LDL, low-density lipoprotein; TG, triglycerides; IL-6, interleukin-6. Mean ± SD, *P < 0.05, **P < 0.01, ***P < 0.001, unpaired t-test; *P_$_ < 0.05, **P_$_ < 0.01, ***P_$_ < 0.001, repeated measure ANOVA between baseline, 1^st^, 2^nd^, and 3^rd^ months.

Despite the average pruritus score of the treatment group being significantly higher than the control group at baseline, supplementation of EPA capsules significantly ameliorated CDKaP, with decreasing average pruritus scores (21.10 ± 6.19, 18.90 ± 5.59, 14.83 ± 5.59, 11.90 ± 7.71) from baseline to the 1st, 2nd, and 3rd month (**Table 3**). In contrast, average pruritus scores (15.70 ± 7.35, 16.80 ± 6.60, 18.03 ± 7.49, and 19.20 ± 8.34) significantly increased in the control group from baseline to the 1st, 2nd, and 3rd month (**Table 3**). Supplementation of EPA capsules also significantly ameliorated pruritus compared to the control group, with a lower pruritus score at 3rd month (**Table 3**). In the examination of skin moisture levels on arms and face, we found that supplementation of soybean oil had no influence on skin moisture, and there were no changes in moisture levels of arms (27.49 ± 15.97, 25.98 ± 10.77, 26.96 ± 15.00, and 25.10 ± 13.88; TDC value) and face (45.13 ± 23.25, 46.53 ± 25.35, 44.78 ± 24.69, and 43.45 ± 24.24; TDC value) at baseline, 1st, 2nd, and 3rd month (**Table 3**). Supplementation of EPA significantly enhanced skin moisture on both arms and face, as well as increased moisture levels on arms (27.49 ± 15.97, 25.98 ± 10.77, 26.96 ± 15.00, and 25.10 ± 13.88; TDC value) and face (45.13 ± 23.25, 46.53 ± 25.35, 44.78 ± 24.69, and 43.45 ± 24.24; TDC value) from baseline to the 1st, 2nd, and 3rd month, respectively (**Table 3**). Supplementation of EPA significantly enhanced the skin moisture level on the face but not on the arms, in comparison with the control group (**Table 3**).

In the examination of inflammatory markers, including CRP and IL-6, we found that EPA supplementation significantly decreased circulating CRP and IL-6 levels in treatment group over a 3-month supplementation period. In contrast, 3-month supplementation with soybean oil did not result in a change in circulating CRP and IL-6 levels (**Table 3**). Additionally, we found that both EPA and soybean oil supplementations did not improve renal function, as well as a non-significant change in circulating CR in both groups (**Table 3**).

In further comparing the lipid profiles following supplementations with EPA and soybean oil, we measured serum CHO, HDL, LDL, and TG (**Table 3**). EPA supplementation led to a significant decrease in CHO concentrations at each respective time point, while soybean oil supplementation did not result in a significant change in CHO concentration (**Table 3**). Additionally, EPA supplementation significantly increased HDL concentrations at each respective time point, whereas soybean oil supplementation did not result in a significant change in HDL concentration (**Table 3**). Neither EPA nor soybean oil supplementation resulted in a change in LDL concentrations over time (**Table 3**). Furthermore, EPA supplementation significantly decreased TG concentrations at each respective time point, whereas soybean oil supplementation did not result in a significant change in TG concentration (**Table 3**).

Although the depression level was higher in the EPA group at baseline, we found that EPA supplementation significantly decreased depression levels from baseline to the first, second, and third months, as well as decreased the BDI score, respectively (P < 0.001, **Table 3**). In contrast, soybean oil supplementation increased the BDI scores from baseline to the first, second, and third months, respectively (P < 0.01, **Table 3**). There were no significant differences between the groups following supplementation, as well as at the 1st month, 2nd month, and 3rd month (**Table 3**).

In further analysis of the correlation between pruritus levels and moisture levels, CRP, IL-6, CHO, CR, HDL, LDL, TG, and BDI scores, we found that pruritus levels and arm moisture levels showed a significantly negative correlation at all time points, including baseline, 1st month, 2nd month, and 3rd month (**Table 4**). Additionally, pruritus levels and face moisture levels also exhibited a significantly negative correlation at the 3rd month (**Table 4**). Serum CRP was positively correlated with pruritus levels at baseline and the 3rd month (**Table 4**). However, there was no significant correlation between pruritus levels and IL-6, CHO, CR, HDL, LDL, TG, and BDI scores at each time point (**Table 4**).

**Table 4:** Correlation of pruritus score against skin moisture levels, inflammatory factors, and lipid profile in treatment group.

	Moisture level (TDC value)		CRP	IL-6	CHO	CR	HDL	LDL	TG	BDI score
	Arms	Face								
**Baseline**	-0.411*	0.061	0.393*	0.048	-0.127	0.291	-0.052	-0.076	-0.192	-0.098
**1^st^ Month**	-0.397*	-0.163	-0.046	-0.137	-0.312	-0.072	0.072	-0.110	-0.118	0.112
**2^nd^ Month**	-0.365*	-0.310	0.010	-0.070	-0.125	0.016	0.080	-0.147	-0.020	0.182
**3^rd^ Month**	-0.427*	-0.451*	0.382*	0.007	-0.086	0.068	0.121	-0.021	0.040	0.324

CRP, c-reactive protein; CR, creatinine; CHO, total cholesterol; HDL, high-density lipoprotein; LDL, low-density lipoprotein; TG, triglycerides; IL-6, interleukin-6. *P <0.05, Pearson’s correlation.

## 4 Discussion

The clinical management of CKDaP remains a significant challenge, especially in ESRD patients and those on dialysis. The complex and unclear mechanism that contributes to pruritus symptoms and unfavorable skin conditions poses a challenge in the development of therapeutic treatments. To date, four major mechanisms including toxin deposition, abnormal nerve conduction, inflammation, and opioid imbalance, have been suggested to contribute to CKDaP (7). Among them, treatment approaches targeting the opioid pathway have been predominantly developed and have yielded promising results (7). Currently, Difelikefalin, a peripherally restricted and selective agonist of κ-opioid receptors, is the first FDA-approved drug for severe CKDaP (35, 36). Although Difelikefalin significantly improves pruritus conditions, adverse effects such as diarrhea, dizziness, vomiting, and gait disturbance were commonly reported (35, 36). While these adverse effects were mostly mild or moderate, up to 9.3% of patients discontinued Difelikefalin treatment due to adverse effects, suggesting a potential limitation for its long-term use in some hemodialysis patients (36). Another κ-opioid receptor agonist, Nalfurafine, has been approved and marketed in Japan and some Asian countries (37, 38). Nalfurafine effectively reduces CKDaP, but it induces adverse effects, mostly insomnia and constipation, which also led to discontinuation (39, 40). Although targeting opioid receptors has yield promising results in ameliorating CKDaP with mostly mild to moderate adverse effects, these effects may lead to discontinuation and suggest some limitations. Therefore, further development and investigation of treatments or interventions are still needed for the management of CKDaP.

Because the pathogenesis of CKDaP has been described as multifactorial, the complexity of mechanical interactions involved in the contribution of pruritus symptoms has led to difficulty in identifying therapeutic targets and developing identical treatment regimens (2, 6). Among these pathogenic mechanisms, chronic inflammation has been suggested as one major contributor to CKDaP (41–43). Higher levels of T-helper 1 cells, CRP, IL-2, and IL-6 were found in maintenance dialysis patients with pruritus compared to those without pruritus6 (44, 45). Immunomodulatory therapies, such as ultraviolet B phototherapy and immunosuppressant, or improvement of dialysis methods showed s reduction in inflammation significantly correlated with decreased intensity of CKDaP (45, 46). Moreover, oral Difelikefalin reduced pruritis and inflammatory biomarkers in patients with atopic dermatitis (47), suggesting that improvement of the systemic inflammatory state is one of the key therapeutic targets for CKDaP. Omega-3 PUFAs are well-known for their anti-inflammatory properties and have been suggested as a supplementation to improve the management of inflammatory diseases (48). EPA has been reported to suppress NF-κB activation, and subsequent expression of proinflammatory cytokines (49). In the present study, we reported that EPA significantly decreased serum CRP and IL-6 following 3 months of supplementation (**Table 3**). However, there was no statistical difference in CRP and IL-6 between groups (**Table 3**). The significantly positive correlation between serum CRP and pruritus score indicates that systemic inflammation is involved in eliciting pruritus (**Table 4**). However, we found no correlation between serum IL-6 and pruritus score (**Table 4**). Further investigation, by increasing the scale of the cohort number or detecting other inflammatory factors such as IL-2, IL-31, and TNFα, that were reported to elicit CKDaP, is needed to address the therapeutic mechanism of EPA supplementation in CKDaP (50).

On the other hand, most patients with CKDaP exhibit abnormal skin pruritogen profiles, characterized by the accumulation of pruritogens including prostaglandins, histamine, cytokines, neuropeptides (such as substance P and nerve growth factor), proteases, and uremic toxins (such as β2-microglobulin) (51). These pruritogens, released by keratinocytes, lymphocytes, mast cells, neurons, or other cells present in the epidermis and dermis, not only elicit pruritus, but also interact with immune cells, resulting in the dysregulation of dermal immunity and exacerbating the severity of pruritus (51, 52). Moreover, xerosis is frequently found in maintenance dialysis patients and has been suggested as a contributing pathogenic factor for CKDaP (5). Although the pathogenesis of uremic xerosis is still unclear, skin inflammation due to increased cutaneous mast cell releasing histamine has been suggested to contribute to uremic xerosis (5). However, the pathogenic role of xerosis in eliciting CKDaP is controversial. Moisturizing the skin in patients with both CKDaP and xerosis has been showed to ameliorate pruritus, but not all maintenance dialysis patients with xerosis suffer from pruritus, suggesting that xerosis may act as an aggravating factor that enhances itching sensation (51). Improving CKDaP in patients with uremic pruritus by moisturizing the skin may play a part in the relief of itchiness. Supplementation of omega-3 PUFAs has been reported to increase skin moisture (21–23). In the present study, we found that EPA supplementation significantly improved skin moisture on both the face and arms over the 3-month study period (**Table 3**). The improvement of skin moisture was not found in the placebo group (**Table 3**). EPA supplementation enhanced skin moisture, especially on the face, in comparison with the placebo group (**Table 3**). The significant negative correlation between skin moisture and pruritus level indicates that improving skin moisture is involved in the amelioration of CKDaP (**Table 4**). Our results show that EPA supplementation ameliorates CKDaP, possibly duo to its multiple effects, including anti-inflammation and improvement of skin moisture.

Depression is prevalent in CKD patients and is associated with increased morbidity and mortality. The prevalence of depression accounted for at least 20% to 30% in CKD patients, reported to be higher than in other chronic disease, such as diabetes mellitus (12% to 18%) and coronary artery disease (15% to 23%) (53–55). Moreover, there are several limitations to treatment in CKD patients with depression symptoms, partly because antidepressant medications are not fully accepted by physicians due to concerns about their efficacy and safety, and partly because high medication burden in CDK patients (54, 55). Other nonpharmacological treatments for depression symptoms, such as psychotherapy, exercise therapy, and cognitive-behavioral therapy, have been explored and studied in patients with ESRD (54). However, the scale of these studies on nonpharmacological treatments in CKD patients with depression is small due to the limited suitability of each treatment for individual patient (54). Further exploration and investigation of both pharmacological and nonpharmacological treatments are needed. In the present study, we found that EPA supplementation significantly decreased the BDI score, whereas the BDI score significantly increased in the placebo group over the course of the supplementation (**Table 3**). However, we found no significant difference in BDI scores between the groups at the end point (3rd month) of the study (**Table 3**). In addition, despite finding that EPA supplementation ameliorated depression levels, the average BDI score still indicated mild to moderate depression in both the placebo and the treatment groups (**Table 3**). Combining EPA supplementation with other pharmacological and nonpharmacological treatments might be necessary to investigate in CKD patients with depression. Furthermore, we found a positive correlation between pruritus and BDI scores (**Table 4**). This result indicates that the severity of pruritus is associated with the prevalence and levels of depression.

Although supplementation of omega-3 PUFAs has been reported to benefit renal function and increase the clearance rate of CR (56, 57), our result did not show any influence on serum CR levels, neither between groups nor over the course of supplementation (**Table 3**). Further examination of cardiovascular parameters, including CHO, HDL, LDL, and TG, revealed that EPA supplementation significantly decreased serum CHO and TG, but this effect was not observed in the placebo group (**Table 3**). Both EPA and soybean oil supplementations did not influence serum LDL levels, neither between groups nor over the course of supplementation (**Table 3**). EPA supplementation showed a mild decrease in serum HDL levels, whereas no significant change in serum HDL levels were observed in the placebo group (**Table 3**). These results indicate a limited efficacy of EPA supplementation on renal function and cardiovascular parameters in maintenance hemodialysis patients.

Age and sex are recognized as factors that can significantly influence various physiological processes, including inflammation, skin health, and mood regulation, all of which are key aspects evaluated in this study (58, 59). Additionally, common comorbidities among hemodialysis patients, such as diabetes mellitus, hypertension, and cardiovascular disease, may also impact the outcomes measured, including pruritus severity, skin moisture, inflammation markers, and depression levels (60, 61). While efforts were made to recruit a homogeneous sample population, the potential influence of these confounding factors cannot be entirely ruled out. In future studies, addressing these confounding variables will be crucial for enhancing the robustness of the findings. Adjusting for age, sex, and comorbidities in the statistical analysis would provide a more comprehensive understanding of the observed outcomes and allow for more accurate interpretations of the results. Moreover, studies with larger sample sizes and more extensive assessments of patient demographics and medical histories could provide deeper insights into the potential interactions and effects of these factors on the outcomes observed in this study.

Despite our results showing that supplementation of EPA improves pruritus, skin dryness, inflammation, and depression in maintenance hemodialysis patients, our study has several limitations. First, the relatively small sample size and short cohort study period limited statistical powder to detect differences, including inflammatory factors, renal function, cardiovascular parameters, and depression between groups. Second, supplementation of other omega-3 PUFA such as DHA and the combination of omega-3 PUFAs could be included for a comparison of efficacy and specificity for each species of omega-3 PUFAs. Third, although using the BDI scale is a commonly validated and frequently used self-report questionnaire to rate depressive symptoms in dialysis patient, other clinically administered assessment such as the Hamilton Depression Rating Scale and Montgomery–Asberg Depression Rating Scale could be concurrently used to increase precision and observe changes in depressive symptoms during the study period. Finally, our study did not assess the dietary habits of patients that might potentially interfere with EPA and soybean oil supplementations.

## 5 Conclusions

Based on the results obtained from our study, it is evident that supplementation with EPA holds significant promise in mitigating various symptoms and complications associated with maintenance hemodialysis patients. Firstly, our findings demonstrate notable improvements in pruritus severity, inflammation markers, skin dryness, and depression symptoms following EPA supplementation. These results highlight the potential of EPA to alleviate multiple symptoms commonly experienced by individuals undergoing maintenance hemodialysis. Moreover, the improvements in skin moisture and decreased severity of pruritus symptoms demonstrates the benefits of EPA supplementation in enhancing skin health and relieving discomfort associated with pruritus. While our study provides valuable insights into the potential therapeutic effects of EPA supplementation, it is essential to acknowledge the limitations of our research, including the small sample size and short duration. Additionally, the inability to control for all potential confounding variables may have influenced the observed outcomes. Moving forward, larger-scale studies with longer follow-up periods and comprehensive control for confounding factors are necessary to validate and extend our findings. Furthermore, exploring the underlying mechanisms through which EPA exerts its therapeutic effects and elucidating its optimal dosage and duration of supplementation are crucial areas for future research. Overall, our study supports the use of EPA supplementation as a potential complementary therapy for improving the health and well-being of maintenance hemodialysis patients.

## Data availability statement

The original contributions presented in the study are included in the article/supplementary material. Further inquiries can be directed to the corresponding author.

## Ethics statement

The study was conducted in accordance with the Declaration of Helsinki and was approved by the Ethics Committee of the Kuang-Tien General Hospital (KTGH 10926; 10 September 2020). The studies were conducted in accordance with the local legislation and institutional requirements. The participants provided their written informed consent to participate in this study.

## Author contributions

TC: Conceptualization, Project administration, Supervision, Writing – original draft, Writing – review & editing. YL: Data curation, Formal analysis, Investigation, Methodology, Writing – review & editing. CW: Data curation, Formal analysis, Investigation, Resources, Writing – review & editing.

## References

[1] Simonsen E Komenda P Lerner B Askin N Bohm C Shaw J. Treatment of uremic pruritus: A systematic review. Am J Kidney Dis. (2017) 70:638–55. doi: 10.1053/j.ajkd.2017.05.018 28720208

[2] Agarwal P Garg V Karagaiah P Szepietowski JC Grabbe S Goldust M. Chronic kidney disease-associated pruritus. Toxins (Basel). (2021) 13:527. doi: 10.3390/toxins13080527 34437400 PMC8402524

[3] Pisoni RL Wikstrom B Elder SJ Akizawa T Asano Y Keen ML. Pruritus in haemodialysis patients: International results from the Dialysis Outcomes and Practice Patterns Study (DOPPS). Nephrol Dial Transpl. (2006) 21:3495–505. doi: 10.1093/ndt/gfl461 16968725

[4] Mettang T Kremer AE. Uremic pruritus. Kidney Int. (2015) 87:685–91. doi: 10.1038/ki.2013.454 24402092

[5] Szepietowski JC Reich A Schwartz RA. Uraemic xerosis. Nephrol Dial Transpl. (2004) 19:2709–12. doi: 10.1093/ndt/gfh480 15328388

[6] Shirazian S Aina O Park Y Chowdhury N Leger K Hou L. Chronic kidney disease-associated pruritus: impact on quality of life and current management challenges. Int J Nephrol Renovasc Dis. (2017) 10:11–26. doi: 10.2147/IJNRD.S108045 28176969 PMC5271405

[7] Verduzco HA Shirazian S. CKD-associated pruritus: new insights into diagnosis, pathogenesis, and management. Kidney Int Rep. (2020) 5:1387–402. doi: 10.1016/j.ekir.2020.04.027 PMC7486142 32954065

[8] Begum R Belury MA Burgess JR Peck LW. Supplementation with n-3 and n-6 polyunsaturated fatty acids: effects on lipoxygenase activity and clinical symptoms of pruritus in hemodialysis patients. J Ren Nutr. (2004) 14:233–41. doi: 10.1016/S1051-2276(04)00134-7 15483784

[9] Ahmad S Dasgupta A Kenny MA. Fatty acid abnormalities in hemodialysis patients: effect of L-carnitine administration. Kidney Int Suppl. (1989) 27:S243–6. 2636665

[10] Dasgupta A Kenny MA Ahmad S. Abnormal fatty acid profile in chronic hemodialysis patients: possible deficiency of essential fatty acids. Clin Physiol Biochem. (1990) 8:238–43. 2129482

[11] Yerlikaya FH Mehmetoglu I Kurban S Tonbul Z. Plasma fatty acid composition in continuous ambulatory peritoneal dialysis patients: an increased omega-6/omega-3 ratio and deficiency of essential fatty acids. Ren Fail. (2011) 33:819–23. doi: 10.3109/0886022X.2011.601831 21793790

[12] Ristic V Tepsic V Ristic-Medie D Perunicic G Rasic Z Postic M. Plasma and erythrocyte phospholipid fatty acids composition in Serbian hemodialyzed patients. Ren Fail. (2006) 28:211–6. doi: 10.1080/08860220600574897 16703792

[13] Friedman AN Moe SM Perkins SM Li Y Watkins BA. Fish consumption and omega-3 fatty acid status and determinants in long-term hemodialysis. Am J Kidney Dis. (2006) 47:1064–71. doi: 10.1053/j.ajkd.2006.03.033 16731302

[14] de Gomez Dumm NT Giammona AM Touceda LA Raimondi C. Lipid abnormalities in chronic renal failure patients undergoing hemodialysis. Medicina (B Aires). (2001) 61:142–6. 11374135

[15] Cobo G Lindholm B Stenvinkel P. Chronic inflammation in end-stage renal disease and dialysis. Nephrol Dial Transpl. (2018) 33:iii35–40. doi: 10.1093/ndt/gfy175 PMC6168801 30281126

[16] Cheng AY Wong LS. Uremic pruritus: from diagnosis to treatment. Diagnostics (Basel). (2022) 12:1108. doi: 10.3390/diagnostics12051108 35626264 PMC9140050

[17] Chen YC Chiu WT Wu MS. Therapeutic effect of topical gamma-linolenic acid on refractory uremic pruritus. Am J Kidney Dis. (2006) 48:69–76. doi: 10.1053/j.ajkd.2006.03.082 16797388

[18] Lahiji AP Mortazavi M Tirani SA Moeinzadeh F Bidaki EZ Naini AE. Omega-3 supplementation improves pruritus in continuous ambulatory peritoneal dialysis patients: A crossover randomized pilot clinical trial. J Res Pharm Pract. (2018) 7:195–9. doi: 10.4103/jrpp.JRPP_18_64 PMC6298140 30622987

[19] Gagnon AL Desai T. Dermatological diseases in patients with chronic kidney disease. J Nephropathol. (2013) 2:104–9. doi: 10.12860/JNP.2013.17 PMC3891143 24475435

[20] Zucker I Yosipovitch G David M Gafter U Boner G. Prevalence and characterization of uremic pruritus in patients undergoing hemodialysis: uremic pruritus is still a major problem for patients with end-stage renal disease. J Am Acad Dermatol. (2003) 49:842–6. doi: 10.1016/S0190-9622(03)02478-2 14576662

[21] Barcelos RC de Mello-Sampayo C Antoniazzi CT Segat HJ Silva H Veit JC. Oral supplementation with fish oil reduces dryness and pruritus in the acetone-induced dry skin rat model. J Dermatol Sci. (2015) 79:298–304. doi: 10.1016/j.jdermsci.2015.06.015 26195090

[22] Lin YL Wang CL Liu KL Yeh CN Chiang TI. Omega-3 fatty acids improve chronic kidney disease-associated pruritus and inflammation. Medicina (Kaunas). (2022) 58:796. doi: 10.3390/medicina58060796 35744059 PMC9229849

[23] Huang TH Wang PW Yang SC Chou WL Fang JY. Cosmetic and therapeutic applications of fish oil's fatty acids on the skin. Mar Drugs. (2018) 16:256. doi: 10.3390/md16080256 30061538 PMC6117694

[24] Mosleh H Alenezi M Al Johani S Alsani A FaIraq G Bedaiwi R. Prevalence and factors of anxiety and depression in chronic kidney disease patients undergoing hemodialysis: A cross-sectional single-center study in Saudi Arabia. Cureus. (2020) 12:e6668. doi: 10.7759/cureus.6668 31976185 PMC6968827

[25] Simoes ESAC Miranda AS Rocha NP Teixeira AL. Neuropsychiatric disorders in chronic kidney disease. Front Pharmacol. (2019) 10:932. doi: 10.3389/fphar.2019.00932 31474869 PMC6707423

[26] Shirazian S. Depression in CKD: understanding the mechanisms of disease. Kidney Int Rep. (2019) 4:189–90. doi: 10.1016/j.ekir.2018.11.013 PMC6365402 30775614

[27] Satti MZ Arshad D Javed H Shahroz A Tahir Z Ahmed MMH. Uremic pruritus: prevalence and impact on quality of life and depressive symptoms in hemodialysis patients. Cureus. (2019) 11:e5178. doi: 10.7759/cureus.5178 31565588 PMC6758963

[28] Ozen N Cinar FI Askin D Mut D. Uremic pruritus and associated factors in hemodialysis patients: A multi-center study. Kidney Res Clin Pract. (2018) 37:138–47. doi: 10.23876/j.krcp.2018.37.2.138 PMC6027816 29971209

[29] Deacon G Kettle C Hayes D Dennis C Tucci J. Omega 3 polyunsaturated fatty acids and the treatment of depression. Crit Rev Food Sci Nutr. (2017) 57:212–23. doi: 10.1080/10408398.2013.876959 25830700

[30] Liao Y Xie B Zhang H He Q Guo L Subramanieapillai M. Efficacy of omega-3 PUFAs in depression: A meta-analysis. Transl Psychiatry. (2019) 9:190. doi: 10.1038/s41398-019-0515-5 31383846 PMC6683166

[31] Okereke OI Vyas CM Mischoulon D Chang G Cook NR Weinberg A. Effect of long-term supplementation with marine omega-3 fatty acids vs placebo on risk of depression or clinically relevant depressive symptoms and on change in mood scores: A randomized clinical trial. JAMA. (2021) 326:2385–94. doi: 10.1001/jama.2021.21187 PMC8693224 34932079

[32] Wani AL Bhat SA Ara A. Omega-3 fatty acids and the treatment of depression: a review of scientific evidence. Integr Med Res. (2015) 4:132–41. doi: 10.1016/j.imr.2015.07.003 PMC5481805 28664119

[33] Dashti-Khavidaki S Gharekhani A Khatami MR Miri ES Khalili H Razeghi E. Effects of omega-3 fatty acids on depression and quality of life in maintenance hemodialysis patients. Am J Ther. (2014) 21:275–87. doi: 10.1097/MJT.0000000000000078 24987942

[34] Gharekhani A Khatami MR Dashti-Khavidaki S Razeghi E Noorbala AA Hashemi-Nazari SS. The effect of omega-3 fatty acids on depressive symptoms and inflammatory markers in maintenance hemodialysis patients: a randomized, placebo-controlled clinical trial. Eur J Clin Pharmacol. (2014) 70:655–65. doi: 10.1007/s00228-014-1666-1 24643636

[35] Fishbane S Jamal A Munera C Wen W Menzaghi F Investigators KT. A phase 3 trial of difelikefalin in hemodialysis patients with pruritus. N Engl J Med. (2020) 382:222–32. doi: 10.1056/NEJMoa1912770 31702883

[36] Fishbane S Wen W Munera C Lin R Bagal S McCafferty K. Safety and tolerability of difelikefalin for the treatment of moderate to severe pruritus in hemodialysis patients: pooled analysis from the phase 3 clinical trial program. Kidney Med. (2022) 4:100513. doi: 10.1016/j.xkme.2022.100513 36039153 PMC9418597

[37] Zhang P Xiang S Liu B Wang X Yang X Ye C. Randomized controlled trial of nalfurafine for refractory pruritus in hemodialysis patients. Ren Fai.l. (2023) 45:2175590. doi: 10.1080/0886022X.2023.2175590 PMC9980412 36856148

[38] Kozono H Yoshitani H Nakano R. Post-marketing surveillance study of the safety and efficacy of nalfurafine hydrochloride (Remitch((R)) capsules 2.5 mug) in 3,762 hemodialysis patients with intractable pruritus. Int J Nephrol Renovasc Dis. (2018) 11:9–24. doi: 10.2147/IJNRD.S145720 29391822 PMC5774492

[39] Kumagai H Ebata T Takamori K Muramatsu T Nakamoto H Suzuki H. Effect of a novel kappa-receptor agonist, nalfurafine hydrochloride, on severe itch in 337 haemodialysis patients: a Phase III, randomized, double-blind, placebo-controlled study. Nephrol Dial Transpl. (2010) 25:1251–7. doi: 10.1093/ndt/gfp588 19926718

[40] Kumagai H Ebata T Takamori K Miyasato K Muramatsu T Nakamoto H. Efficacy and safety of a novel k-agonist for managing intractable pruritus in dialysis patients. Am J Nephrol. (2012) 36:175–83. doi: 10.1159/000341268 22868684

[41] Lu PH Wang JY Chuo HE Lu PH. Effects of uremic clearance granules in uremic pruritus: A meta-analysis. Toxins (Basel). (2021) 13:702. doi: 10.3390/toxins13100702 34678995 PMC8540647

[42] Kimmel M Alscher DM Dunst R Braun N Machleidt C Kiefer T. The role of micro-inflammation in the pathogenesis of uraemic pruritus in haemodialysis patients. Nephrol Dial Transpl. (2006) 21:749–55. doi: 10.1093/ndt/gfi204 16249205

[43] Schricker S Heider T Schanz M Dippon J Alscher MD Weiss H. Strong associations between inflammation, pruritus and mental health in dialysis patients. Acta Derm Venereol. (2019) 99:524–9. doi: 10.2340/00015555-3128 30673107

[44] Rastogi A Fishbane S Lerma E. Difelikefalin for the treatment of moderate-to-severe pruritus associated with chronic kidney disease on hemodialysis. Expert Rev Clin Pharmaco.l. (2023) 16:387–400. doi: 10.1080/17512433.2023.2197209 37010031

[45] Ko MJ Peng YS Wu HY. Uremic pruritus: pathophysiology, clinical presentation, and treatments. Kidney Res Clin Pract. (2023) 42:39–52. doi: 10.23876/j.krcp.21.189 35545226 PMC9902728

[46] Malekmakan L Tadayon T Pakfetrat M Mansourian A Zareei N. Treatments of uremic pruritus: A systematic review. Dermatol Ther. (2018) 31:e12683. doi: 10.1111/dth.12683 30141218

[47] Guttman-Yassky E Facheris P Da Rosa JC Rothenberg-Lausell C Del Duca E David E. Oral difelikefalin reduces moderate to severe pruritus and expression of pruritic and inflammatory biomarkers in subjects with atopic dermatitis. J Allergy Clin Immunol. (2023) 152:916–26. doi: 10.1016/j.jaci.2023.06.023 37453614

[48] Simopoulos AP. Omega-3 fatty acids in inflammation and autoimmune diseases. J Am Coll Nutr. (2002) 21:495–505. doi: 10.1080/07315724.2002.10719248 12480795

[49] Balic A Vlasic D Zuzul K Marinovic B Bukvic Mokos Z. Omega-3 versus omega-6 polyunsaturated fatty acids in the prevention and treatment of inflammatory skin diseases. Int J Mol Sci. (2020) 21:741. doi: 10.3390/ijms21030741 31979308 PMC7037798

[50] Schricker S Kimme M. Unravelling the pathophysiology of chronic kidney disease-associated pruritus. Clin Kidney J. (2021) 14:i23–31. doi: 10.1093/ckj/sfab200 PMC8702819 34987780

[51] Molina P Ojeda R Blanco A Alcalde G Prieto-Velasco M Areste N. Etiopathogenesis of chronic kidney disease-associated pruritus: putting the pieces of the puzzle together. Nefrologia (Engl Ed). (2023) 43:48–62. doi: 10.1016/j.nefroe.2023.03.015 37173258

[52] Wong LS Yen YT Lee CH. The implications of pruritogens in the pathogenesis of atopic dermatitis. Int J Mol Sci. (2021) 22:7227. doi: 10.3390/ijms22137227 34281281 PMC8269281

[53] Hedayati SS Yalamanchili V Finkelstein FO. A practical approach to the treatment of depression in patients with chronic kidney disease and end-stage renal disease. Kidney Int. (2012) 81:247–55. doi: 10.1038/ki.2011.358 PMC3258342 22012131

[54] Hedayati SS Finkelstein FO. Epidemiology, diagnosis, and management of depression in patients with CKD. Am J Kidney Dis. (2009) 54:741–52. doi: 10.1053/j.ajkd.2009.05.003 PMC3217258 19592143

[55] Shirazian S Grant CD Aina O Mattana J Khorassani F Ricardo AC. Depression in chronic kidney disease and end-stage renal disease: similarities and differences in diagnosis, epidemiology, and management. Kidney Int Rep. (2017) 2:94–107. doi: 10.1016/j.ekir.2016.09.005 29318209 PMC5720531

[56] Lauretani F Maggio M Pizzarelli F Michelassi S Ruggiero C Ceda GP. Omega-3 and renal function in older adults. Curr Pharm Des. (2009) 15:4149–56. doi: 10.2174/138161209789909719 PMC2863302 20041816

[57] Hu J Liu Z Zhang H. Omega-3 fatty acid supplementation as an adjunctive therapy in the treatment of chronic kidney disease: a meta-analysis. Clinics (Sao Paulo.). (2017) 72:58–64. doi: 10.6061/clinics/2017(01)10 28226034 PMC5251198

[58] Lavretsky H Newhouse PA. Stress, inflammation, and aging. Am J Geriatric Psychiatry. (2012) 20(9):729–33. doi: 10.1097/JGP.0b013e31826573cf PMC3428505 22874577

[59] Duvetorp A Mrowietz U Nilsson M Seifert O. Sex and age influence the associated risk of depression in patients with psoriasis: A retrospective population study based on diagnosis and drug-use. Dermatology. (2021) 237(4):595–602. doi: 10.1159/000509732 PMC8315676 32927456

[60] Cha J Han D. Health-related quality of life based on comorbidities among patients with end-stage renal disease. Osong Public Health Res Perspect. (2020) 11(4):194–200. doi: 10.24171/j.phrp.2020.11.4.08 PMC7442444 32864310

[61] Wu YH Hsu YJ Tzeng WC. Physical activity and health-related quality of life of patients on hemodialysis with comorbidities: A cross-sectional study. Int J Environ Res Public Health. (2022) 19(2):811. doi: 10.3390/ijerph19020811 35055633 PMC8775483

